# The body sends a signal: Perspectives on interoception

**DOI:** 10.1371/journal.pbio.3003517

**Published:** 2025-11-14

**Authors:** Lucas K. Smith, on behalf of the PLOS Biology staff editors

**Affiliations:** 1 Public Library of Science, San Francisco, California, United States of America; 2 Public Library of Science, Cambridge, United Kingdom

## Abstract

Research into brain–body communication is being reinvigorated as the field unites under the umbrella of ‘interoception’. This Editorial highlights four Perspectives that show the breadth of this emerging field.

## Teaser

Research into brain–body communication is being reinvigorated as the field unites under the umbrella of “interoception.” In this issue of *PLOS Biology*, four Perspectives show the breadth of this emerging field, highlighting how interoceptive pathways might be used to promote health.

How are you feeling right now? If you take a moment, you might notice cues from your body—your heartbeat or breathing, a gurgling in your stomach, an ache in your neck—which may contribute to your answer. Indeed, signals from your body are constantly being sensed and integrated by the brain, in a process termed “interoception” [1]. A racing heart or rapid breathing might contribute to feelings of anxiety. Signals from our gut might make us more likely to snack on a sweet. Pro-inflammatory signals in response to a viral infection may make us lethargic. Whether we are aware of these signals or not, they influence many core aspects of what make us ourselves, shifting our emotions, motivation, reflexes, and decision-making.

Interoceptive signals originate from all biological systems to influence homeostatic functioning [2]. The study of these types of brain–body communication has a long [3], and often siloed history [2]. However, in recent years, this field has seen a wave of momentum as neuroscience becomes increasingly interdisciplinary, and as researchers apply a suite of advanced tools, developed for the study of the central nervous system, to interrogate the peripheral nervous system. Efforts to bring the distinct lines of research together under the umbrella of “interoception” [1,2] seek to garner this momentum, which promises to accelerate the pace of research, as diverse communities share insights and tools.

In this issue of *PLOS Biology*, we highlight a few of the exciting topics within interoception research with a mini-collection of opinionated Perspectives on hepatic [4], gut [5], and cardiorespiratory [6] interoception. We also include a forward-looking Perspective that explores how insights from this field might be harnessed to inform “Whole Person Health,” and which lays out steps the field can take to realize this goal [7]. There is a clear consensus between these Perspectives in support of the importance of brain–body communication as a regulator of physiology and behavior. However, these pieces also identify and discuss challenges within this field and highlight the need for more research to further define interoceptive mechanisms and their functional relevance.

The need for additional mechanistic studies is highlighted in Young-Hwan Jo’s Perspective on hepatic interoception [4], in which he discusses the relevance of liver-derived signals to metabolic homeostasis and mood. Jo discusses recent and historical evidence indicating that the liver is innervated by both vagal sensory and parasympathetic cholinergic inputs, and that this facilitates bi-directional communication that can influence metabolism, feeding behavior, and anxiety. However, there are discrepancies in the literature about the extent and nature of hepatic innervation, with some in the field questioning whether the hepatic parenchyma is actually directly innervated by vagal afferents [8]. This debate highlights the need to develop new tools for the study of visceral signaling in general, and to clarify the precise nature of liver–brain communication.

Complementing the idea that signals from the body influence metabolism, in their Perspective, Sung, and Small [5] discuss the relevance of gut interoception for the obesity epidemic. After we eat, post-oral signals from the gut transmit feedback about the nutrient and energy value of the meal. This subliminal mechanism influences food reward and future eating behavior independently of the conscious perception of food (i.e., taste) [9]. Sung and Small discuss how these interoceptive feedback signals can become blunted by the regular consumption of unhealthy diets. They argue that the shift in interoception, rather than changes to the conscious pleasure of food, constitutes the major mechanism by which current dietary trends promote overconsumption and obesity.

The emerging consensus that interoceptive signals influence homeostasis and that disruption of interoceptive mechanisms can be deleterious, raises the exciting prospect that interoceptive processes can also be targeted to help restore homeostasis and improve human health and well-being. To that end, Kluger and colleagues discuss recent findings linking cardiorespiratory rhythms and signals to perception, emotional processing, memory, and motor activity, and discuss the therapeutic implications of this work [6]. Given that respiration is under autonomous control, breath training may represent a useful tool in a number of therapeutic contexts, including the treatment of panic attack disorders. Despite the promise of interoception-targeting therapies, Kluger and colleagues caution that these interventions need to be well grounded. They argue for more mechanistic and validation studies to ensure that “interoception” does not become a buzzword.

Finally, in their Perspective [7], Chen and Langevin argue that interoception may represent a key mechanism contributing to Whole Person Health, an index that encompasses factors including health and quality of life, social connections, diet and activity, stress, and sleep. They lay out their vision for a research framework that will aid the development of interoception-targeted therapies and argue that the field needs to develop and apply new tools to measure, map, monitor, and modulate interoceptive signals. Collectively, these efforts will provide a deeper understanding of interoception that can be leveraged to promote well-being.

While we have only covered a small subset of the exciting research being done in the field of interoception, we hope this cross-section of opinions offers an interesting snapshot of this vibrant field. As researchers from different backgrounds coalesce to share tools and insights, the field is well-positioned to unlock fundamental insights into what makes us all tick. As a leader in open access publishing for over 20 years, publishing exciting research across biological scales and at the interface of different fields, at *PLOS Biology* we are keenly interested in research that advances understanding into brain–body communication. We hope to help this field continue to grow and develop as it continues to uncover how we feel and function.

## Acknowledgments

The PLOS Biology staff editors are Nonia Pariente, Ines Alvarez-Garcia, Roland Roberts, Christian Schnell, Joanna Clarke, Richard Hodge, Lucas Smith, Taylor Hart, Melissa Vazquez Hernandez, and Ankiit Ahluwalia.
